# Systematic identification of latent disease-gene associations from PubMed articles

**DOI:** 10.1371/journal.pone.0191568

**Published:** 2018-01-26

**Authors:** Yuji Zhang, Feichen Shen, Majid Rastegar Mojarad, Dingcheng Li, Sijia Liu, Cui Tao, Yue Yu, Hongfang Liu

**Affiliations:** 1 Division of Biostatistics and Bioinformatics, University of Maryland Marlene and Stewart Greenebaum Comprehensive Cancer Center, Baltimore, Maryland, United States of America; 2 Department of Epidemiology and Public Health, University of Maryland School of Medicine, Baltimore, Maryland, United States of America; 3 Division of Biomedical Statistics and Informatics, Department of Health Sciences Research, Mayo Clinic, Rochester, Minnesota, United States of America; 4 School of Biomedical Informatics, University of Texas Health Science Center at Houston, Houston, Texas, United States of America; 5 Department of Medical Informatics, School of Public Health, Jilin University, Changchun, Jilin, China; King Abdullah University of Science and Technology, SAUDI ARABIA

## Abstract

Recent scientific advances have accumulated a tremendous amount of biomedical knowledge providing novel insights into the relationship between molecular and cellular processes and diseases. Literature mining is one of the commonly used methods to retrieve and extract information from scientific publications for understanding these associations. However, due to large data volume and complicated associations with noises, the interpretability of such association data for semantic knowledge discovery is challenging. In this study, we describe an integrative computational framework aiming to expedite the discovery of latent disease mechanisms by dissecting 146,245 disease-gene associations from over 25 million of PubMed indexed articles. We take advantage of both Latent Dirichlet Allocation (LDA) modeling and network-based analysis for their capabilities of detecting latent associations and reducing noises for large volume data respectively. Our results demonstrate that (1) the LDA-based modeling is able to group similar diseases into disease topics; (2) the disease-specific association networks follow the scale-free network property; (3) certain subnetwork patterns were enriched in the disease-specific association networks; and (4) genes were enriched in topic-specific biological processes. Our approach offers promising opportunities for latent disease-gene knowledge discovery in biomedical research.

## Introduction

In recent decades, a vast amount of biomedical research has been conducted to investigate disease classifications, health records, clinical trials, and adverse event reports that can be utilized to establish links between disease and genes, in order to identify novel treatments for diseases [1]. This effort provides an unprecedented opportunity to extract phenotype-genotype associations, which plays an important role toward the eventual development of a comprehensive, relational, multi-dimensional “data translator” integrating multiple types of existing data sources [2].

Biomedical literature is one of the richest and most reliable information resources and extracting association information from literature is critical for scientists to explore potential associations among different biomedical concepts, such as diseases and genes [3]. To facilitate and expedite such an investigation process, natural language processing (NLP) has been extensively applied to automatically extract association information from biomedical literature, such as the Semantic MEDLINE database (SemMedDB) [4, 5]. However, due to the huge volume of data with complex associations and noises, it is still challenging to discover knowledge from literature. In addition, current disease classification is mostly done through phenotypic observations while ignoring the underlying molecular and pathophysiological information. Therefore, to tackle these issues, a novel integrative informatics framework needs to be designed.

Latent Dirichlet Allocation (LDA) is a generative computational model aiming to explain sets of observations by unobserved variable groups [6]. Recently, LDA has been widely used to uncover underlying semantic associations among biomedical concepts embedded in medical databases and public domain in the informatics field. For instance, Arnold et al [7] applied LDA to identify clinically significant topics using case-based patients’ notes. Angues et al [8] employed an unsupervised LDA method to prioritize clinical dialogues for visualizing shared content in communication. Wang et al [9] proposed BioLDA to identify complex biological relationships in literature. Wu et al [10] proposed a probabilistic Kullback-Leibler (KL) distance based on LDA to rank the gene-drug associations in biomedical literature. Bisgin et al [11, 12] and Bian et al [13, 14] used LDA in the drug repositioning research. LDA-based approaches have also been used for information retrieval such as interpretation of MeSH terms in literature [15], diversity ranking of genomics information retrieval in microbial studies [16, 17], and MeSH-indexing with labeled LDA [18]. For example, Chen et al [17] proposed to identify functional groups in microbial gene catalogue using LDA by considering functional elements (e.g., taxonomic levels, indicators of gene orthologous groups, and KEGG pathway mappings) as words and each functional group as topics in the LDA modeling. Their experimental results showed that topic modeling could effectively cluster functional elements into highly interpretable groups.

In recent years, network-based computational approaches have become one of the major analytical and visualization tools to extract informative content from high dimensional data and reduce noise among disease and gene associations in biomedical research [19]. Applications of these approaches include drug repositioning [20, 21], disease gene prioritization [22–24], and identification of disease relationships [25, 26]. For instance, Hu and Agarwal [27] created a human disease-drug network based on genomic expression profiles collected from the GEO database, in which 170,027 interactions between diseases and drugs were considered significant. These expression-based associations between diseases and drugs could serve as future research directions. Bauer-Mehren et al [28] developed a comprehensive disease-gene association network by integrating associations from several sources that covers different biomedical aspects of diseases. The results indicated a highly shared genetic origin of human diseases. To systematically analyze disease-drug-gene relationships, Daminelli et al [29] proposed a network-based approach to predict novel drug-gene and drug-disease associations by completing incomplete bi-cliques in the network. This approach holds great potential for discovery of novel disease mechanisms and drug repositioning. For a detailed review of network-based approaches, please refer to a series of review articles [19, 30–32]. One of such network approaches enables us to analyze heterogeneous networks by decomposing them into statistically significant recurring subnetworks, called network motifs (NMs) [33]. They are the smallest basic functional and evolutionarily conserved units in various types of biological networks. Network motifs are usually considered as significant sub-patterns representing the backbone of the network by forming larger network modules with specific functional roles. In our previous study [34], we developed a network motif-based approach to investigate vaccine-related disease-drug-gene network, demonstrating that a combinatorial analysis using literature knowledgebase, semantic technology, and network approach is able to reveal latent knowledge critical to biomedical research and public health and generate testable hypotheses for future experimental verification.

**Fig 1:**
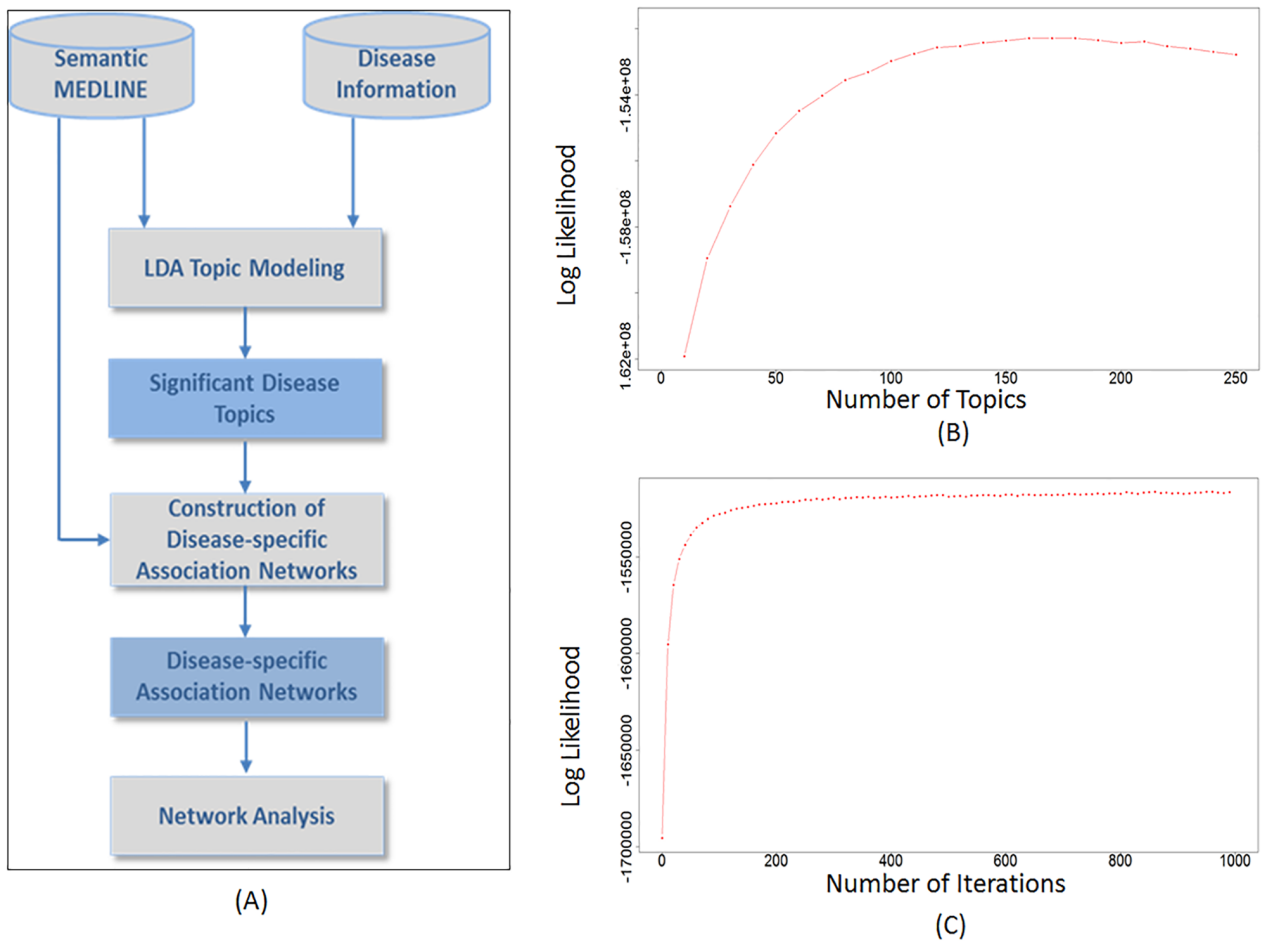
(A) Overview of the proposed approach. (B) The Log likelihood score across different number of topics. (C) The Log likelihood score across different iterations.

In this study, we proposed an integrative informatics framework that leverages LDA and network analysis to facilitate novel knowledge discovery using disease-gene association information extracted from literature. Literature mining will enable us to stay current. The ability of LDA to represent distributed semantics embedded in data will enable us to group diseases based on associated molecular and pathophysiological level information. Further reducing the dimensionality and noises through network analysis can expedite the discovery. Specifically, our approach is able to detect latent disease topics with semantic granularity and discover potential important disease mechanisms from the literature with minimum noises. First, we applied an LDA-based modelling approach to group 7,039 diseases into 160 optimal disease topics based on 146,245 disease-gene associations recorded in SemMedDB Version 25. Based on the diseases and genes involved in each disease topic, we constructed a network for each disease topic and investigated latent novel disease mechanisms based on a series of network properties. Specifically, in our case study topic of Alzheimer’s Disease (AD), we examined the properties of the association network by investigating both overall network properties such as node degree distribution, and local network structure called network motifs. The genes involved in each association network were also analyzed by gene set enrichment analysis. The overall approach is illustrated in Fig 1(A). Our results demonstrate that (1) the LDA-based approach is able to group related diseases into same disease topics based on their high-dimensional yet sparse associations with genes; (2) the disease-specific association network follows the scale-free network property, in which hub nodes are rich in diseases and genes closely related with each other; (3) significant network motif patterns can be detected in the disease-specific networks indicating novel yet latent disease mechanisms; and (4) genes in the association network are significantly enriched in biological processes and canonical pathways highly involved in hub diseases.

## Results

### LDA-based modelling revealed diverse disease topics and their associated genes

From SemMedDB Version 25, we extracted 146,245 disease-gene associations between 7,039 diseases and 10,921 genes from titles and abstracts of over 25 million PubMed articles. Diseases were identified by semantic type (e.g. “*dsyn*” or “*neop*”) and genes were identified by gene terms approved by HGNC (https://www.genenames.org/). Based on our LDA modelling on these associations, we assembled these 7,039 diseases into 160 optimal disease topics. The optimal number of disease topics was determined by the log likelihood score defined in the Method section. As shown in Fig 1(B), the highest log likelihood score was obtained when LDA grouped the diseases into 160 topics. In addition, in 1000 iterations of the LDA algorithm, the curve of log likelihood score was convergent at topic number of 160 (Fig 1(C)), indicating that is a reasonable optimum number of topics.

**Fig 2:**
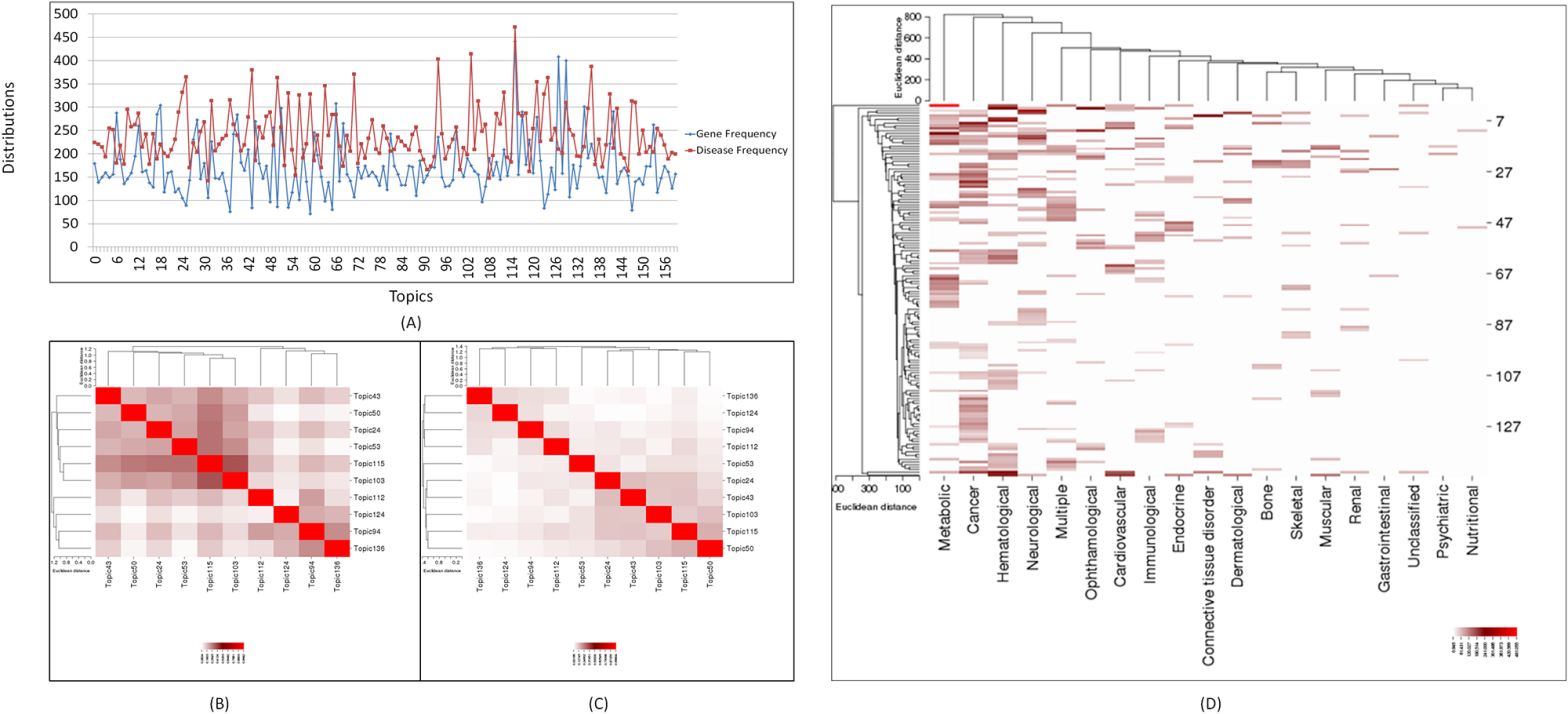
(A) Distribution of diseases and genes across 160 optimal disease topics. (B) The heatmap of cosine similarity for top 10 topics presented at disease level. (C) The heatmap of cosine similarity for top 10 topics presented at gene level. (D) Overall Distribution of 146 LDA Topics on 19 Human Disease Network Categories in Goh et al.

In our LDA result, each topic can be represented as a group of diseases associated with the same groups of genes. It can also be viewed as a group of genes involved in similar diseases. We investigated the distribution of diseases and genes across the 160 topics, in which the gene distribution represented the number of genes assigned to each topic with non-zero proportion, and the disease distribution represented the number of diseases contributing to each topic. Topics 115, 127, 129, 66 and 18 are the top 5 topics involving largest number of genes (i.e., 441, 408, 400, 308, and 304 genes respectively), while Topics 123, 65, 147, 37 and 59 are the 5 topics with the smallest number of genes (i.e., 83, 80, 79, 76 and 71 respectively). Similarly, Topics 115, 103, 94, 136 and 43 are top the 5 topics containing the largest number of diseases (i.e., 472, 414, 403, 387 and 380 diseases respectively), while Topics 146, 119, 55, 108 and 31 are 5 topics containing the smallest number of diseases (i.e., 163, 162, 154, 148 and 142 respectively). To examine the distributions between genes and diseases in same topics, we overlaid both distributions in Fig 2(A). We observed that the number of genes was not necessarily correlated with that of diseases in same topics. In most cases, topics contain a larger number of diseases than genes, indicating that many diseases may share common genes. However, a few topics (e.g., Topic 119 with 162 diseases and 230 genes) contain a relative small number of diseases while containing many shared genes, suggesting that diseases in these topics are very complex and have many genes involved.

To further explore the extent to which each disease topic contains the disease-gene association information in our dataset, we ranked 160 topics based on their normalized posterior probabilities, top 10 of which were presented in S1 Table. Topic 115 had the highest posterior probability of 0.02346, indicating that LDA assigned the largest number of genes to Topic 115. In other words, Topic 115 contained the largest number of genes involved in different diseases.

We also investigated the similarities among disease topics based on their containing genes and diseases using the cosine similarity approach. Cosine similarity [35] is commonly used on two non-zero vectors to measure the cosine of the angle between them in order to quantify the similarity between vectors. Here we compared each topic with all the others based on their diseases and genes, aiming to explore the divergences among topics generated by LDA. We conducted a pair-wise comparison of the distribution of cosine similarity values based on diseases and genes for each topic-topic pair. The cosine similarity is used in the positive space, whose value range is between 0 and 1. Therefore, we partitioned [0, 1] into ten interval groups in each of which the frequency of topic-topic pairs was shown in S2 Table. No topic pairs showed significantly high similarities, suggesting that LDA is capable to group similar diseases into same groups. In general, topics shared a higher level of similarity at disease level than gene level, suggesting that even phenotypically similar diseases might be grouped into different groups based on their associations with different groups of genes representing distinct biological processes.

We then took a closer look at the similarity among the top 10 topics at disease and gene level respectively (Fig 2(B) and 2(C)). These 10 topics were divergent at gene level. The highest similarity was observed between Topic 50 and 115 (similarity score is 0.15), which shared 30 genes including *pik3ca*, *neurl1*, *hpse*, *neu1*, and *birc5*. Consistent with the results of overall cosine similarity measurement, the similarities of top 10 topics were higher at disease level (average value is 0.26) than at gene level (average value is 0.146). These observations suggested that although there are overlapping genes and diseases among topics, our LDA process was able to generate distinct disease groups based on the disease-gene associations embedded in SemMedDB.

### Comparison of disease topics with disease categories in other works

To further evaluate the extent of the coverage of disease topics generated by our LDA analysis, we compared the 160 topics with existing disease categories annotated by Goh et al [36]. We downloaded the human disease network file “diseasesome.gexf” (https://exploringdata.github.io/info/human-disease-network/), and extracted 3,926 disease-gene associations between 784 unique diseases and 638 unique genes. These 784 diseases were grouped into 22 categories, including Bone, Cancer, Cardiovascular, Connective tissue disorder, Dermatological, Developmental, Ear-Nose-Throat, Endocrine, Gastrointestinal, Hematological, Immunological, Metabolic, Multiple, Muscular, Neurological, Nutritional, Ophthamological, Psychiatric, Renal, Respiratory, Skeletal, and Unclassified. We extracted the diseases in our 160 LDA-derived disease topics and mapped them onto these 22 categories. To capture a broader mapping, we did the mapping by checking both disease names and their synonyms. Using the method proposed by Frick et al [37], we considered diseases to be similar if they share a common ancestor within three generations. We used SNOMED-CT[38], a comprehensive and systematically organized ontology of medical terms, for disease similarity calculation. We found that 19 out of 22 disease categories can be mapped to 146 topics, except Developmental, Ear-Nose-Throat, and Respiratory. The heatmap in Fig 2(D) represents the overall distribution between 146 disease topics in our results and 19 disease categories in Goh et al [36]. We found that Cancer has the highest overlap level with the LDA disease topics, i.e., the Cancer category in Goh et al has observable overlaps with 73 LDA topics. Metabolic, Hematological, and Neurological also have relatively higher overlap with LDA topics, i.e., they have observable overlaps with 49, 46, and 39 LDA topics respectively. However, Gastrointestinal, Unclassified, Psychiatric, and Nutritional have the least coverage of diseases contained in each topic, which indicates that SemMedDB has relatively fewer disease-gene associations related to these categories. A detailed list of diseases contained in each topic is presented in S1 File.

To better describe relationships between topics and disease categories in Goh et al, we also listed top topics that contributed the most diseases for each category as shown in Table 1. We found that Metabolic has the most diseases in Topic 39, and Neurological also has a significant number of diseases in Topic 34. In addition, Hematological, Ophthamological, Connective tissue disorder, Cancer shared relatively bigger groups of diseases with Topic 109, 52, 54, and 107 respectively. Although Gastrointestinal category does not overlap with many topics, it shared 133 diseases with Topic 152, indicating that Gastrointestinal category has a closer association with this topic. Similarly, Nutritional, Psychiatric, and Unclassified have closer association with Topics 2, 145, and 39 respectively.

**Table 1:** Topics with the most diseases mapped on Human Disease Network Categories.

Disease Category in Goh et al	Mapped LDA Topic	# Mapped Diseases
Bone	96	141
Cancer	107	209
Cardiovascular	159	184
Connective tissue disorder	54	238
Dermatological	1	116
Endocrine	154	135
Gastrointestinal	152	133
Hematological	109	296
Immunological	33	129
Metabolic	39	482
Multiple	27	123
Muscular	114	140
Neurological	34	333
Nutritional	2	56
Ophthamological	52	242
Psychiatric	145	77
Renal	38	120
Skeletal	41	110
Unclassified	39	75

To further evaluate the 146,245 disease-gene associations extracted from SemMedDB, we compared them with disease-gene associations annotated by the Online Mendelian Inheritance in Man (OMIM) [39] knowledge base. For each disease topic, we calculated the distribution of disease-gene association coverage as described in the Methods section. In total, 159 topics have disease-gene associations annotated by the OMIM. The only exception is Topic 37. In general, the average coverage across the 159 topics is 17.8%. We listed top 10 topics with the highest OMIM disease-gene association coverage in Table 2, in which Topic 123 held the highest coverage as 32.3%. A detailed list containing the percentage of disease-gene associations shared by each LDA topic and OMIM is presented in S2 File.

**Table 2:** Top 10 LDA topics containing most OMIM disease-gene associations.

LDA Topic	Percentage of Disease-Gene Associations overlapped with OMIM
123	32.3%
149	29.2%
23	27.3%
76	27.2%
30	26.7%
112	23.8%
117	23.7%
13	22.7%
94	22.6%
135	22.4%

### Evaluation of disease topics at disease level

**Fig 3:**
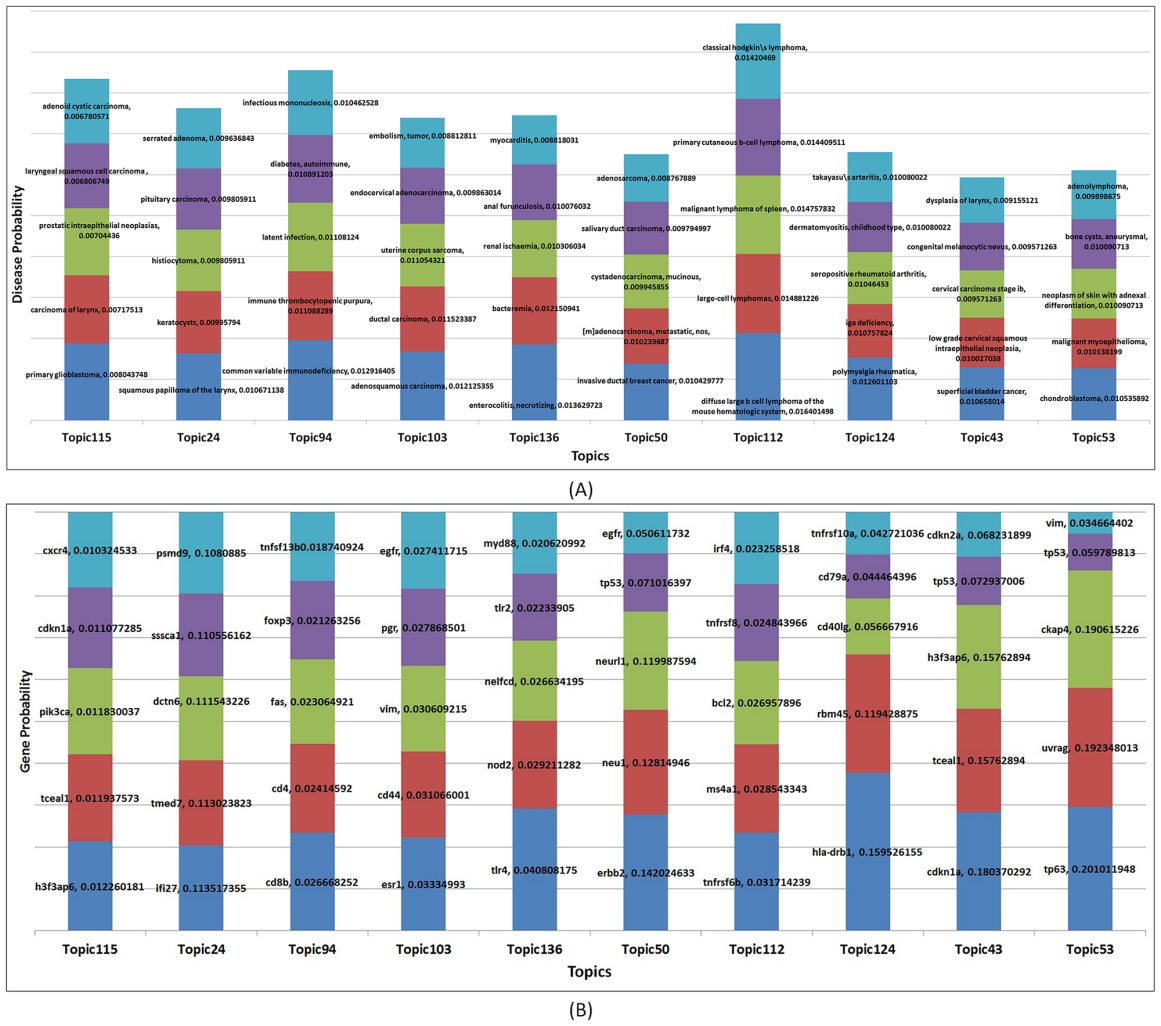
(A) Top 10 topics and their corresponding top 5 diseases based on probabilities. (B) Top 10 topics and their corresponding top 5 genes based on probabilities. For both figures, color blue, red, green, purple, and cyan represent top 1 to 5 diseases/genes respectively.

To investigate the detailed composition of disease topics at disease level, we first examined the distribution of the top 5 diseases in terms of their probabilities for top 10 disease topics (Fig 3(A)). The most representative diseases in these topics were quite diverse, including primary glioblastoma, squamous papilloma of the larynx, common variable immunodeficiency, adenosquamous carcinoma, enterocolitis necrotizing, invasive ductal breast cancer, diffuse large B cell lymphoma of the mouse hematologic system, polymyalgia rheumatic, superficial bladder cancer, and chondroblastoma.

**Fig 4:**
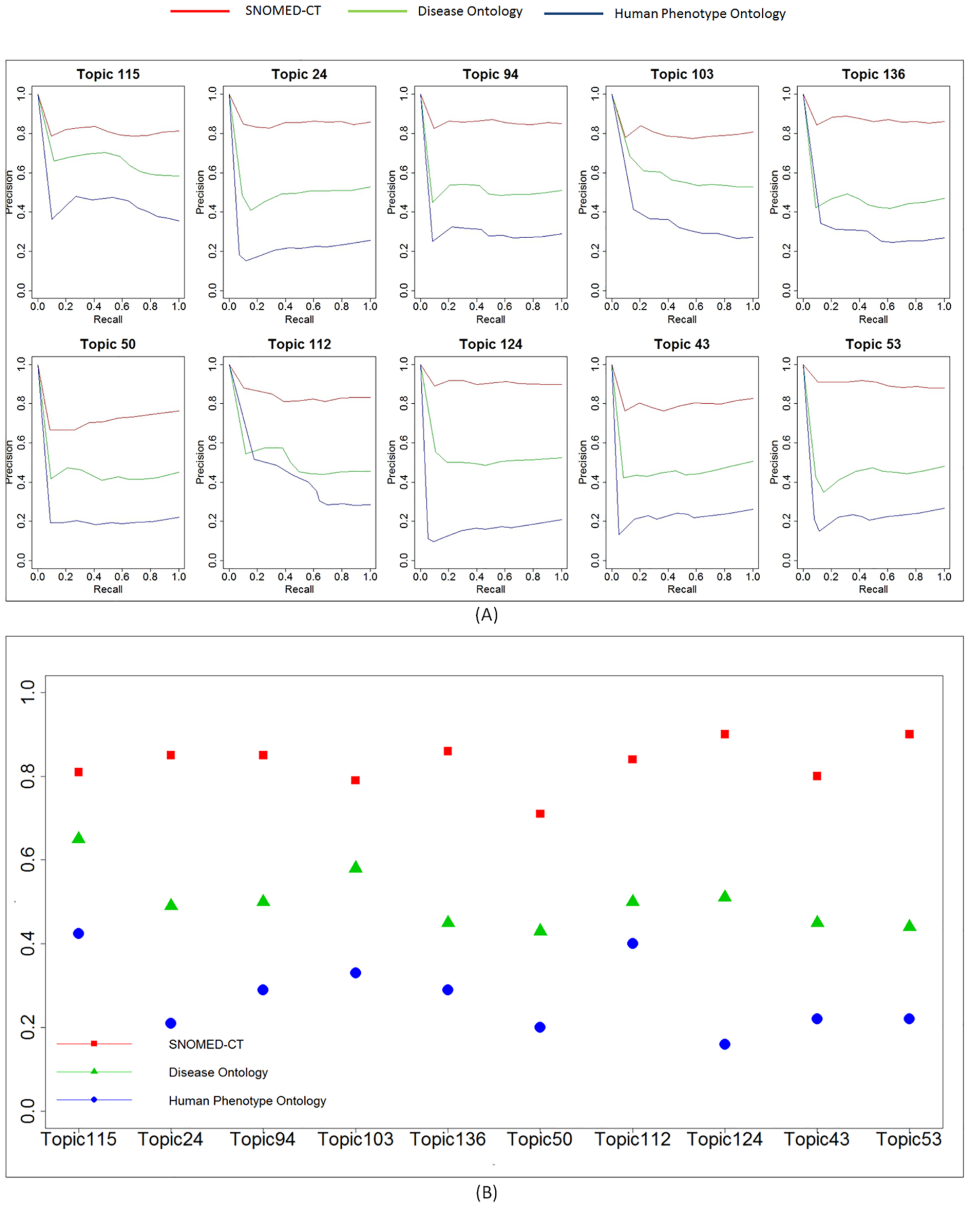
(A) The precision recall curve for top ten topics annotated by three independent disease ontologies. (B) Area under curve (AUC) score for top 10 topics using three independent disease ontologies.

To further systematically evaluate the similarity of diseases involved in each disease topic, we adopted three widely used disease ontologies (i.e., SNOMED-CT [38], Disease Ontology (DO) [40] and Human Phenotype Ontology (HPO) [41]) to investigate the semantic similarities between diseases within each topic as well as across topics. We defined that two diseases are related if they share the same ancestor nodes within three levels of the ontology hierarchy [37]. S3 Table lists the statistics of three ontologies provided by BioPortal [42]: SNOMED-CT contains the largest number of classes, properties and children, DO holds the smallest number of classes with a medium size of properties and children, and HPO maintains a medium number of classes with the smallest size of properties and children. First, we annotated diseases in top 10 topics with three different ontologies. For SNOMED-CT, we found all SNOMED codes including their synonyms for each disease in our dataset. For DO and HPO, we extracted all UMLS CUI along with their synonyms for each disease. S4 Table presents the annotation results by three ontologies. For each topic, SNOMED-CT provides the highest coverage of annotation, DO has the second highest coverage of annotation, and HPO has the lowest coverage of annotation. We applied the information retrieval metrics to evaluate if one disease has higher similarity with other diseases in the same topic than ones annotated by same ontology terms which is considered as one of the gold standards. The purpose was to investigate if each LDA topic can indeed group similar diseases together. We employed the precision-recall curve, one of the widely adopted information retrieval metrics. Specifically, the precision was defined as the percentage of diseases that has at least one similar disease in the topic, and the recall was defined as the percentage of diseases that have similar pairs in both LDA topic and ontologies. For all diseases in top 10 topics, we divided them into 10 folds and calculated the precision and recall for each fold to measure the trade-off between precision and recall. The precision-recall curve for each of top 10 topics was presented in Fig 4(A). In most topics, SNOMED-CT achieved the best performance, DO the second, and HPO the worst except for Topic 136 and 112, with recall ranging from 0 to 0.2. This result was consistent with their coverage difference of disease terms. We also measured the area under curve (AUC) for each ontology annotation (Fig 4(B)) and obtained the same results, i.e., SNOMED-CT had the highest AUC scores while HPO had the lowest AUC scores across the top 10 topics due to its low annotation coverage of disease terms in our study. The average AUC score derived from SNOMED-CT was around 0.8, suggesting that our LDA grouping is consistent with independent disease ontology knowledge derived by the biomedical community. To give a comprehensive evaluation of ontology matching for LDA topics, we computed the AUC score for all 160 topics as shown in S1 Fig. We found that evaluation results are pretty consistent with which conducted for top 10 topics, indicating that SNOMED-CT has the highest matching performance, Disease Ontology is the second highest one and HPO achieved the lowest performance.

### Evaluation of disease topics at gene level

To explore whether there were some dominant genes allocated in each disease topic, we examined the top 5 genes in terms of their probabilities for top 10 disease topics (Fig 3(B)). We observed that genes *h3f3ap6*, *ifi27*, *cd8b*, *esr1*, *tlr4*, *erbb2*, *tnfrsf6b*, *hla-drb1*, *cdkn1a* and *tp63* occupied the largest proportion in the top 10 topics respectively. We then calculated the LDAKL score as described in Methods section for the top two genes within each topic and pair of top two genes across the top 10 topics (S5 Table). The LDAKL scores of top two genes in same topics were much smaller than that of two genes from different topics, suggesting that the LDA grouping were capable of grouping similar genes into distinctive disease topics.

### Network analysis of disease-gene association networks

To explore novel yet latent disease-gene association(s) within each disease topic, for each of the top ten disease topics, we constructed a disease-specific association network by extracting associations involving these disease terms. As shown in Table 3, each disease topic focused on some specific disease categories. The association statistics were also presented in Table 3, in which each association network contains thousands of nodes (i.e., disease, and genes) and edges (i.e., associations between nodes). Since these associations are usually high-dimensional yet noisy, it is impractical for domain experts to manually investigate these associations. To address this problem, we investigated these association networks with a series of network properties such as hub nodes and degree distribution. The overall results were listed in Table 3, suggesting that these networks share the scale-free network properties as other biological networks. A table containing the statistics and network properties for all 160 disease topics was presented in S3 File. In the following three case studies, we used three disease topics (i.e., Alzheimer’s Disease, asthma-lymphoma, and lymphoma) to demonstrate that a more thorough network-based informatics approach can expedite the identification of novel disease-gene associations and interpret them in a biologically meaningful way.

**Table 3:** Statistics of top ten disease topics.

Topic ID	Hub Disease (node degree)	Number of Nodes	Number of Associations	Network Diameter	Characteristic Path Length
**115**	carcinoma, non-small-cell lung (219)	608	10,895	5	2.55
	squamous cell carcinoma (210)				
	neoplasm metastasis (194)				
**24**	chronic b-cell leukemias (42)	459	2,957	6	3.01
	cancer of rectum (41)				
	liver neoplasms (38)				
**94**	Asthma (86)	398	2,971	6	2.82
	lymphoma, large-cell, diffuse (85)				
	chronic lymphocytic leukemia (77)				
**103**	endometrial carcinoma (58)	330	2,293	6	2.83
	epithelial ovarian cancer (52)				
	malignant neoplasm of endometrium (50)				
**136**	rheumatoid arthritis (76)	378	2,259	6	2.94
	inflammatory bowel diseases (68)				
	inflammatory disorder (63)				
**50**	salivary gland neoplasms (17)	244	1,058	7	2.71
	prostatic intraepithelial neoplasias (14)				
	mucinous neoplasm (14)				
**112**	Lymphoma (97)	377	1,883	8	3.02
	lymphoma, large-cell, diffuse (85)				
	chronic lymphocytic leukemia (77)				
**124**	celiac disease (29)	265	892	7	3.03
	Sarcoidosis (22)				
	graves disease (21)				
**43**	malignant neoplasm of skin (16)	231	984	7	2.57
	dysplastic nevus (13)				
	carcinoma in situ of uterine cervix (12)				
**53**	uterine cervical neoplasms (14)	240	799	6	2.69
	mouse pancreatic intraepithelial neoplasia-2 (12)				
	endometrial adenocarcinoma (11)				

#### Case study 1: Alzheimer’s disease topic

Among all 160 disease topics, AD has non-zero proportion in 55 of them. We focused on Topic 61, the most representative AD topic based on its proportion, to further illustrate the AD related mechanisms. Specifically, we applied a network-based analytical approach to dissect and prioritize significant biomedical concepts and associations in this network. The diseases with highest node degrees include not only AD, but also Parkinson’s disease, neurodegenerative disorders, and amyotrophic lateral sclerosis, all of which have been shown to have significant associations with AD (S6 Table). There are also other highly connected diseases with less known associations with AD (e.g., *tardive dyskinesia*), which could serve as promising future directions in AD research. This case study clearly demonstrates the superiority of network-based approach in inferring indirect associations among diseases in a disease topic generated using LDA modelling. This association network also showed a scale-free network property, in which certain diseases and genes act as “hubs” (S2 Fig). We then analyzed the local network structure by performing a network motif analysis on the AD network as described in our previous work [43]. Overall, there were three significant network motifs in the AD-specific association network (S7 Table). The finding that certain network motifs are statistically enriched in the association network compared to random networks of same network topology indicates that these network motifs represent underlying biological specificity that could not be found in other networks.

To explore the pathways and biological processes the genes in the AD association network are enriched in, we performed a gene set enrichment analysis using the Ingenuity Pathway Analysis (IPA) tool (see a complete gene list in S4 File). Table 4 listed the top ranked canonical pathways and networks enriched in the genes, most of which have been proven to be associated with AD. For instance, since AD is the common cause of dementia, it is not surprising to see that the Huntington’s disease signaling pathway is enriched in the topic. Because both AD and Parkinson’s disease are neurodegenerative (i.e., brain cells (neurons) become damaged and die during the course of the disease), we also found that Parkinson’s signaling pathway is enriched in the topic. In addition, we observed that the G Protein-Coupled Receptors (GPCRs) pathway is enriched in the AD topic. There have been many studies demonstrating the link between GPCRs and AD, whereas the effect of GPCRs on AD progress is yet to be further explored given its complexities [44]. The mutations in Amyloid Precursor Protein (APP) have been associated with the pathogenesis of Alzheimer’s disease in many recent studies [45–48]. We found four enriched pathways involving APP: Mitochondrial Dysfunction, WReelin Signaling in Neurons, Neuroprotective Role of THOP1 in AD, and Amyloid Processing (S5 File). Some of these pathways can serve as potential future research directions for the AD research community. There are also other significant pathways and networks that are not well known to be associated with AD (see a complete pathway list in S5 File), which could serve as potential future research directions in AD research.

**Table 4:** A list of enriched diseases and disorders associated with genes in the AD association network.

Canonical Pathways	-log(p-value)	Ratio	Molecules
Huntington′s Disease Signaling	8.95	0.06	BDNF,CREBBP,TBP,NGF,TGM2,HDAC6,GRM5,AKT1,HDAC3,ATP5B,KL,HTT,DLG4,DCTN1,SNCA
G-Protein Coupled Receptor Signaling	6.5	0.05	GRM5,HTR2C,FYN,AKT1,GRK2,KL,CREBBP,PRKAR1B,HTR1A,DRD3,DRD2,ADORA2A,HTR2A
Neuropathic Pain Signaling In Dorsal Horn Neurons	5.37	0.08	GRM5,NTRK2,GPR37,BDNF,KL,GRIN2D,PRKAR1B,ELK1
Parkinson′s Signaling	5.21	0.25	GPR37,PARK7,PARK2,SNCA
Mitochondrial Dysfunction	4.74	0.05	SOD2,ATP5B,PARK7,LRRK2,HTRA2,PARK2,SNCA,APP,PINK1
Neurotrophin/TRK Signaling	4.37	0.07	AKT1,NTRK2,BDNF,KL,CREBBP,NGF
PEDF Signaling	4.28	0.07	SOD2,AKT1,BDNF,KL,NGF,ELK1
Serotonin Receptor Signaling	4.23	0.09	HTR2C,GCH1,SLC6A4,HTR1A,HTR2A
Dopamine Receptor Signaling	4.09	0.07	GCH1,COMT,PRKAR1B,DRD3,DRD2,SLC6A3

P Value: B and H multiple testing corrected p-values; Ratio: number of molecules in a given pathway that meet cut criteria, divided by total number of molecules that make up that pathway.

#### Case study 2: Lung cancer topic

In this case study, we investigated the top disease topic focusing on lung cancer, a leading cause of cancer death in men and women in the United States [49]. The assembled disease-gene association network consists of 608 nodes (i.e., 180 diseases and 428 genes) and 10,895 associations between them. The mostly highly connected diseases include "*carcinoma*, *non-small-cell lung*", “*squamous cell carcinoma*”, and “*neoplasm metastasis*”. The network motif analysis found same significant three-node network motifs in this lung cancer-specific association network as the ones in Case Study 1. The gene set enrichment analysis suggested that these genes are statistically enriched in many cancer signalling pathways, such as p53 signalling, pancreatic adenocarcinoma signalling, and prostate cancer signalling. Most of the top ranked canonical pathways and networks enriched in these genes have been proven to be associated with lung cancer (S6 File). These results suggested that 1) the lung cancer topic shares similar network properties as other disease-gene association networks, where important diseases were prioritized through network analysis, and 2) genes allocated in the topic were enriched in biological processes that can serve as potential research focuses in lung research.

#### Case study 3: Asthma-lymphoma topic

In this case study, we selected the disease topic of asthma and lymphoma. There have been a few studies discussing potential association between asthma and lymphoma, implying that the common cause and progression of the two diseases relates to some common imbalance of the immune system [50, 51]. Our assembled disease-gene association network of Topic 94 suggested that these two diseases are indeed associated with a large number of common diseases and genes (i.e., 180 diseases and 279 genes) through 10,895 associations. The mostly highly connected diseases include "*asthma*", “*lymphoma*, *large-cell*, *diffuse*”, and “*chronic lymphocytic leukemia*”. Similarly, three same network motifs were identified in the asthma-lymphma topic. The enriched pathways in the 279 genes include the Th1 and Th2 Activation Pathway [52, 53], Crosstalk between Dendritic Cells and Natural Killer Cells [54], and Altered T Cell and B Cell Signaling [55–57], among many cancer and immune related biological processes and functions (S7 File). Another interesting finding is enriched lupus-related biological processes in this asthma-lymphoma topic. Although lupus is not a highly connected disease term, many genes are annotated by many biological processes involved in lupus annotated by the independent IPA enrichment analysis tool. Other significant pathways and genes that are not well known to be associated with lupus can serve as future directions.

## Discussions and conclusions

To address the issues of semantic granularity and inherent noises brought by high-dimensional disease-gene association data mined from literature, we proposed an integrative analytical framework which combines LDA and network analysis to facilitate latent disease-gene association discovery and provide insights into the relationship between molecular and cellular processes and diseases. Specifically, we applied LDA modelling to identify significant disease topics based on thousands of disease-gene associations mined from literature. Within each disease topic, we reconstructed and dissected a disease topic-specific association network to explore novel yet latent disease mechanisms by network properties as well as independent biological knowledge. The analysis of disease-specific association networks, exemplified by the AD disease topics, demonstrated that our approach is capable to prioritize significant association patterns and prominently expedite novel yet latent disease knowledge discovery. To our knowledge, our approach is the first attempt to integrate both topic modelling and network decomposition techniques for the discovery of novel disease mechanisms, allowing for high-dimensional reduction, noise removal, and nonlinear latent association inference among multiple biomedical concepts rather than pairwise associations.

As a community-based knowledge resource, ontology based classification is also able to detect disease-disease and gene-disease associations. However, the major difference between ontology and LDA is that ontology only contains explicit semantic information and our proposed framework enables the use of empirical distributed semantics to assist the exploration of associations. Meanwhile, novel association discovery highly depends on real-time knowledge update while there is a latency in capturing the latest information in ontology-based approaches [58]. The interpretation of “novel” genes can be difficult due to the fact that some genes may not be “novel” since they have already been published in literature. For example, OMIM doesn’t include the association between the *p*.*E318G* variant and Alzheimer’s disease (AD) [59], while it is reported in the literature. Specifically, in our AD case study, we conducted an additional experiment to compare the number of associations identified by LDA and OMIM. In total, there are 544 AD associated genes identified from SemMedDB. Our LDA approach was able to recover 144 of them among top 10 topics. Meanwhile, there were only 46 AD-related genes annotated by OMIM. This comparison suggests our framework can be used as a complementary data-driven approach to mine latent disease-gene associations from large collections of literature (e.g., PubMed) in order to detect latent novel associations and help enrich existing ontology.

There have been many discussions between LDA and unsupervised clustering approaches. The main reason that we adopted LDA is that LDA is in fact a unique bi-clustering approach [60]. In our study, we employed LDA to cluster genes based on their co-occurrences in the same document(s), which can reflect which genes are semantically closer. Meanwhile, LDA clusters documents based on the gene distributions within them. Other clustering methods such as k-means, can only consider one type of similarity measurement during the grouping process. Topic modelling approaches such as LDA can consider one gene assigned to multiple disease topics based on their similarities to other genes in the same topic, i.e., LDA is a mixture model. Different from usual soft clustering, we consider both document similarity and gene similarity in the LDA process. Furthermore, LDA is also a robust generative Bayesian modelling approach, which specifically fits the big data analysis. The robustness comes from partially that LDA adopts conjugate distribution, such as Dirichlet and multinomial to build models. These features are unique to LDA not seen in many unsupervised methods.

In this work, we focused on disease-gene associations in SemMedDB. Besides disease-gene associations, there are other types of disease-related associations that we can obtain, such as disease-drug associations. Our approach can be easily adopted to dissect such complicated and heterogeneous associations in the future, leading to other biomedical applications such as drug repositioning. In addition, our LDA modelling is able to remove the strict reliance on a given ontology. Instead of learning only from the keywords which map directly to an ontology class, LDA can use a vocabulary more tailored to the association data on which it is trained. Additionally, LDA can form associations from multiple types of information at once, in which topics may include a mixture of genetic or phenotypic information (e.g., genes) as well as any other clinically relevant characteristics (e.g., drugs). It is especially useful that the output of the model is interpretable and can be easily inspected. Our results suggest that the proposed LDA process is able to better differentiate topics by genes than by diseases, which also reflect the essence of topic modelling.

One limitation of our current study is that SemMedDB now only contains disease-gene associations based on the co-occurrence relationship. Therefore, even if some diseases do not have close biomedical associations with each other, they may be still clustered together based on their co-occurrences with other biomedical terms. Since the focus of this study is to demonstrate the capacity of LDA in grouping closely related diseases and SemMedDB has ~77% prediction accuracy of associations [61], we expect similar false positive discovery rate in our study. To address such challenges, we propose to extend and refine the proposed approach in a few directions. First, instead of using the uninformed priors for alpha, we can provide a prior from the dataset itself. Second, instead of assigning a fixed number of topics beforehand, we will employ a hierarchical Dirichlet process to automatically find the best number of topics. Third, we can calculate the distances between topics so that these topics with closer distances can be merged. We believe that all three refinements will lead to better and more accurate grouping in the LDA process. We also plan to integrate our current dataset with other data resources such as omics data and OMIM knowledge base. For instance, interactive LDA, in which manual reviews can be incorporated into iterations of topic generations, could be a promising framework. Also, by including complementary gene-disease association data resources, we anticipate to increase the prediction accuracy in future research. We expect to extend our work in several related research topics, including (1) integration of additional supervised information (e.g., key words for PubMed abstracts) to make LDA generate more controllable and interpretable topics [62–64]; (2) integration of more comprehensive association databases among disease, drug, and gene (e.g., HPRD [65] and DrugBank [66]) to construct more complete base association networks; (3) a framework to automatically extract such disease-specific association network so that such analysis can be extended to each disease topic; (4) additional network-based investigation of the relationships among disease, drug, and gene at other network levels such as module subnetwork identification; and (5) investigation on possible ways to improve the network by assigning weights or confidence values to different types of associations or associations from different sources.

## Materials and methods

### Retrieval of disease-gene association data from SemMedDB

Semantic MEDLINE Database (SemMedDB) is a repository of semantic predications (i.e., subject-predicate-object triples) extracted from the titles and abstracts of all PubMed citations [5]. In this study, we used SemMedDB Version 25, which contains more than 84 million predications (i.e., associations) between concepts extracted from titles and abstracts of over 25 million PubMed indexed [67]. Since we focused on the investigation of disease-gene associations, we developed a preprocessing framework (S3 Fig) to extract disease-gene associations from the sentence predication table in SemMedDB. First, we used the semantic type filtering strategy to retain associations relevant to diseases only (i.e., only predications involving semantic types *dsyn (Disease or Syndrome)* or *neop (Neoplastic Process)* were kept). Second, we used the gene symbols approved by HGNC[68] to retain associations relevant to genes only. Through this filtering process, we were able to generate the list of disease-gene associations recorded in SemMedDB.

### Disease grouping with latent dirichlet allocation

In this study, each disease was considered as a document containing its associated gene(s) recorded in SemMedDB. We used these disease-gene associations as the input data for LDA analysis. S8 File lists some examples of disease-gene associations we used for LDA analysis.

The LDA hierarchical Bayesian generative process is shown in S4 Fig, in which the big plate represents a collection of documents, the middle small plate represents one document *p*, node *c* refers to one gene, and arrows denote the conditional probability dependencies. For a topic *z*_*i*_, we denote the proportion of a gene *c* allocated in *z*_*i*_ as *c*_*i*_. Each gene *c*_*i*_ has a probability $\phi_{c_{i}}^{z_{i}}$ in topic *z*_*i*_, where i refers to the index of each gene, *c*_*i*_ is a scalar to represent the ith gene, and *z*_*i*_ is a vector to represent the topic of ith gene. The uniqueness of LDA is that it places symmetric Dirichlet priors on both $\theta_{z_{i}\;}^{p_{j}\;}$ and $\phi_{c_{i}}^{z_{i}}$, with $\theta_{z_{i}\;}^{p_{j}\;}\sim \;Dirichlet(\alpha )$ and $\phi_{c_{i}}^{z_{i}}\sim \;Dirichlet(\beta )$. The sparsity of distributions can be controlled with hyper-parameters α and β. The above generative process can be summarized as:

$$c_{i}|z_{i},\phi_{c_{i}}^{z_{i}}\;\sim \;Polynomial\left(\phi_{c_{i}}^{z_{i}}\right),\;i=1,\ldots ,C$$ (1)

$$\phi_{c_{i}}^{z_{i}}\;\sim \;Dirichlet\left(\beta \right),\;\;z_{i}=1,\;\ldots ,\;K$$ (2)

$$z_{i}|\theta^{p_{j}}\;\sim \;Polynomial\left(\theta^{p_{j}}\right),\;\;i=1,\ldots ,\;C$$ (3)

$$\theta^{p_{j}}\sim \;Dirichlet\left(\alpha \right),\;p_{j}=1,\;\ldots ,\;P$$ (4)

where *p*_*j*_ represents the *j*^th^ disease (i.e., document), and *z*_*i*_ denotes the topic of the *i*th gene (i.e., word *c*_*i*_). Each gene in the vocabulary *c*_*i*_ ∈ *V* = [*c*_1_, *c*_2_, …, *c*_*C*_] is assigned to each latent topic variable *z*_*i*_. Given a topic *z*_*i*_ = *k*, the expected posterior probability ${\hat{\theta }}^{p_{j}}$ of topic mixings of a given disease *p*_*j*_ and the expected posterior probabilities ${\hat{\phi }}_{c_{i}}^{z_{i}}$ of gene *c*_*i*_ are calculated as below:

$${\hat{\phi }}_{c_{i}}^{z_{i}}=\frac{n_{c_{i}k}+\beta }{\sum_{j=1}^{C}n_{c_{j}k}+C\beta }$$ (5)

$${\hat{\theta }}^{p_{j}}=\frac{n_{p_{j}k}+\alpha }{\sum_{k=1}^{K}n_{p_{j},k}+P\alpha }$$ (6)

where $n_{c_{i}k}$ is the count of *c*_*i*_ in topic *k*, and $n_{p_{j},k}$ is the count of topic *k* in the disease *p*_*j*_. In the LDA process, the values of hyper-parameters *α* and *β* need to be determined beforehand: the former controls the disease distributions, while the latter controls the gene distributions. The higher *α* is, the more similar the diseases are within same disease topics. Similarly, the higher *β* is, the more similar the topics are according to gene distributions. The optimal values of *α* and *β* can be obtained through the grid search. In this study, we set *α* as 0.1 of topics while *β* as 0.01 according to LingPipe LDA implementation (http://alias-i.com/lingpipe/demos/tutorial/cluster/read-me.html).

To obtain the posteriors in the LDA analysis, we used collapsed variational Bayesian inference (CVB) because of relatively large number of topics in our study and its computational efficiency [69]. After we obtained the posteriors, we calculated the log-likelihood of the whole collection of documents by integrating all the latent variables.

### Determination of optimal disease topics

The number of topics was determined heuristically by examining a range of topic number with fixed step size and choosing the one with the highest log likelihood value indicating the optimal topic number as described in Griffiths et al [70]. The log likelihood is defined as

$$p\left(c|z\right)=\Pi_{t=1}^{T}\left[\int_{\phi_{z_{t}}}p(c\left|,\phi_{z_{t}})p(\phi_{z_{t}}\right|z_{t})d\phi_{z_{t}}\right]=\left[\frac{\Gamma {\left(C\beta \right)}^{T}}{\Gamma {\left(\beta \right)}^{C}}\right]\cdot \Pi_{t=1}^{T}\frac{\prod_{c_{i}}\Gamma \left(n_{t}{}^{c_{i}}+\beta \right)}{\Gamma \left(n_{t}{}^{\left(\cdot \right)}+C\beta \right)}$$ (7)

### Evaluation of gene similarity

The KL divergence is used to evaluate how similar two genes associated with the same disease are. Due to its directionality, we defined a symmetric version of KL divergence, called LDAKL, as in Eq (8):

$$\begin{array}{ll} LDAKL\left(c_{i},c_{j}\right) & =KL\left(c_{i},c_{j}\right)+KL\left(c_{j},c_{i}\right)\\ & =\Sigma_{t=1}^{T}p\left(c_{i}|t\right)\text{log}\left(\frac{p\left(c_{i}|t\right)}{p\left(c_{j}|t\right)}\right)+\Sigma_{t=1}^{T}p(c_{j}|t)\text{log}\left(\frac{p(c_{j}|t)}{p(c_{i}|t)}\right) \end{array}$$ (8)

### Similarity between topics

We evaluated the topic similarity using the cosine similarity of their contained genes and covered diseases for each pair, respectively. Eq (9) calculated the cosine similarity between topics, where *X*_*l*_ and *Y*_*l*_ represent the components of gene/disease vector *X* and *Y* for any two topics, m indicates the total number of components in *X* and *Y*.

$$Cosine\;Similarity=\frac{\sum_{l=1}^{m}X_{l}Y_{l}}{\sqrt{\sum_{l=1}^{m}X_{l}^{2}}\;\sqrt{\sum_{l=1}^{m}Y_{l}^{2}}}$$ (9)

### Calculation of the disease-gene association coverage

The disease-gene association coverage (DGAC) was calculated as

$$DGAC\left(A,B\right)=\frac{\left|A\cap B\right|}{\left|A\right|}$$ (10)

where the sets A and B represent disease-gene associations found in each topic and OMIM respectively.

### Calculation of precision and recall

The precision and recall rate of disease category using independent ontology knowledge is calculated by the following equations. Specifically, for any given diseases A and B in the same topic, they are considered a similar pair if A and B are also considered similar in ontology. Each ontology is considered as a gold standard containing annotated diseases in ontology.

$$Precision=\frac{Number\;of\;annotated\;diseases\;that\;have\;similar\;pairs\;found\;in\;ontology\;}{Number\;of\;extracted\;diseases}$$ (11)

$$Recall=\frac{Number\;of\;annotated\;diseases\;that\;have\;similar\;pairs\;found\;in\;ontology\;}{Number\;of\;gold\;standard\;diseases\;}$$ (12)

### Network property of disease-specific association network

For each disease topic, a disease-gene association network was reconstructed consisting of diseases and their associated genes. The disease-gene associations were integrated into a bipartite disease-gene association network. In this network, nodes represent biomedical concepts (i.e., diseases or genes), and edges between nodes represent associations between two nodes (e.g., association between diseases and genes). The important diseases/genes were identified by their significant higher node degree compared to other diseases/genes in the same network. The Cytoscape tool [71] was used to analyze and visualize the network.

### Network motif analysis

In this study, we focused on three-node network motif identification for this disease- gene network since larger size network motifs (number of nodes > 3) are usually composed of three-node network motifs in most cases [43]. All connected subnetworks containing three nodes in the interaction network were collated into isomorphic patterns, and the number of times each pattern occurred was counted. The number of occurrences was at least five (the default setting of the algorithm) for each pattern to be considered as a candidate network motif. In addition, statistical significance test was performed by generating 1000 randomized networks and computing the fraction of randomized networks in which the pattern appeared at least as often as in the interaction network [72]. The *z* score is calculated using the following equation:

$$Z=\frac{N_{real}-<N_{rand}>\;}{\sigma_{rand}}$$ (13)

where *N*_*real*_ is the number of times one three-node subnetwork was detected in the real network, < *N*_*rand*_ > is the mean number of times this subnetwork was detected in 1000 randomized networks, and *σ*_*rand*_ is the standard deviation of the number of times this subnetwork was detected in randomized networks. The *p* value of a motif is the number of random networks in which it occurred more often than in the original networks, divided by the total number of random networks. By default, a pattern with *p*≤0.05 was considered statistically significant. This network motif discovery procedure was performed using the FANMOD tool [73].

### Gene set enrichment analysis

The gene set enrichment analysis in one disease topic was conducted using the IPA tool (http://www.ingenuity.com). This tool maps and generates enriched putative networks and pathways based on the manually curated knowledge database of pathway interactions extracted from the literature. Pathways were ranked by significance scores that measured the probability of genes included in the pathway by chance. Specifically, a hypergeometric test was applied to the genes involved in one pathway against the whole gene knowledge base manually curated in IPA. The canonical pathways were ranked by the adjusted P value. An adjusted *p* value less than 0.01 was used as cut off to select enriched canonical pathways.

## Supporting information

S1 Fig Precision Recall Area Under Curve (PRAUC) for 160 topics. (TIF)

S2 Fig The distribution of node degree for the AD-specific association network (Topic 61). (TIF)

S3 Fig The preprocessing framework to extract disease-gene associations from SemMedDB. (TIF)

S4 Fig The LDA hierarchical Bayesian generative process. (TIF)

S1 File A detailed list of diseases contained in each topic. (XLSX)

S2 File A detailed list containing the percentage of disease-gene associations shared by each LDA topic and OMIM. (XLSX)

S3 File The the statistics and network properties for all 160 disease topics. (XLSX)

S4 File A complete gene list used for gene set enrichment analysis. (XLSX)

S5 File A list of all significant pathways and networks enriched in the AD topic (P value < 0.05). (XLSX)

S6 File A list of top ranked canonical pathways and networks enriched in lung cancer (P value < 0.05). (PDF)

S7 File A list of enriched pathways in the 279 genes in the asthma-lymphoma topic (P value < 0.05). (PDF)

S8 File Some example disease-gene associations used for LDA analysis. (XLSX)

S1 Table Top 10 topics based on their normalized posterior probability. (DOC)

S2 Table Distribution of disease-based nad gene-based similarity for topic-topic association. (DOC)

S3 Table Statistics of three disease ontologies. (DOC)

S4 Table Overview of the annotation results with three ontologies. (DOC)

S5 Table LDKAL score between top 10 topics (optimal values in bold). (DOC)

S6 Table List of diseases with highest node degree in the AD association network. (DOC)

S7 Table Statistics of significant network motifs. (DOC)
