# Supplementary figures and images for: Systematic identification of latent disease-gene associations from PubMed articles
Source: PLoS One. 2018 Jan 26;13(1):e0191568. doi: 10.1371/journal.pone.0191568 (PMC5786305; doi:10.1371/journal.pone.0191568)

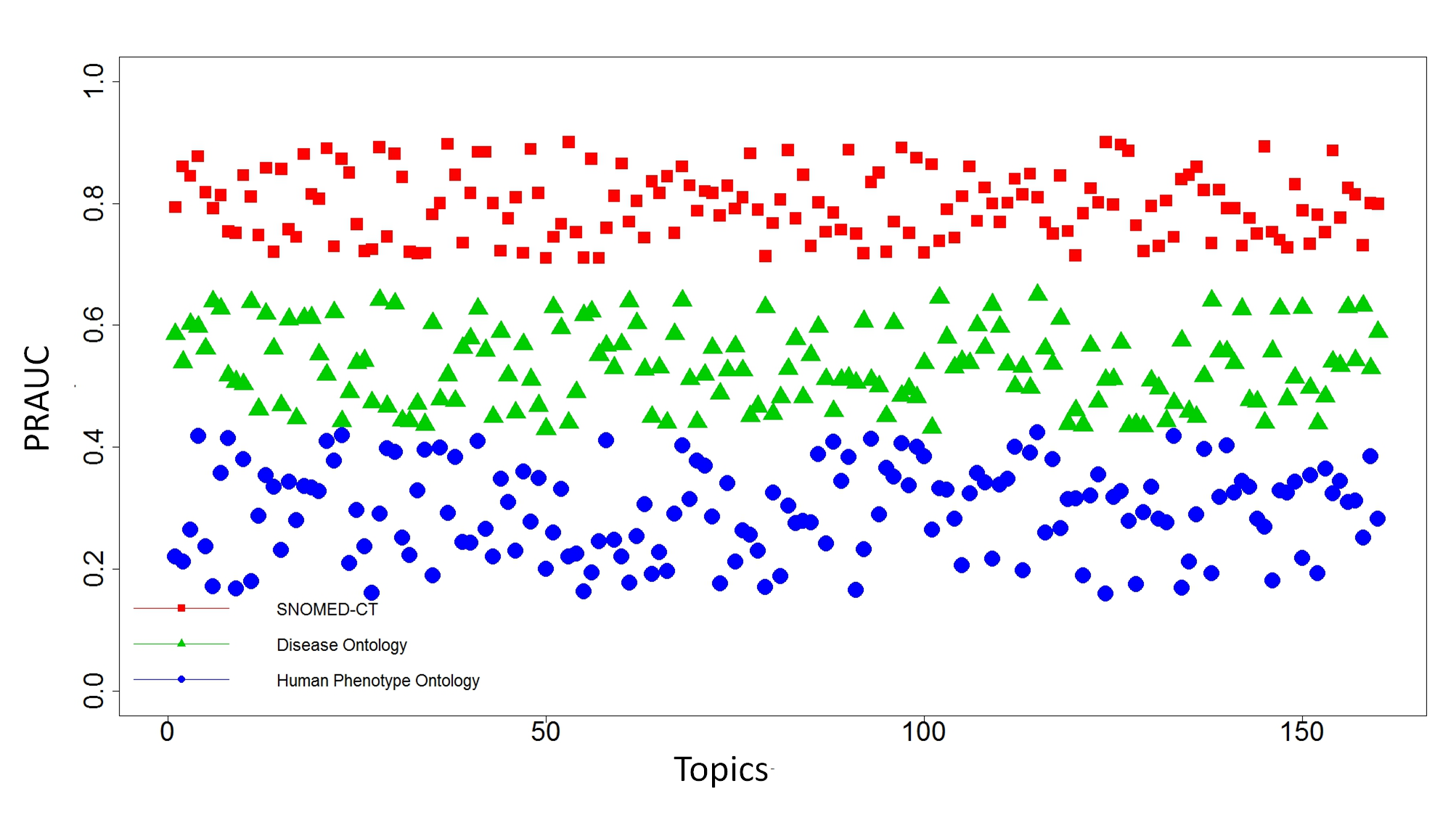

Supplement: S1 Fig — (TIF) [file pone.0191568.s001.tif]

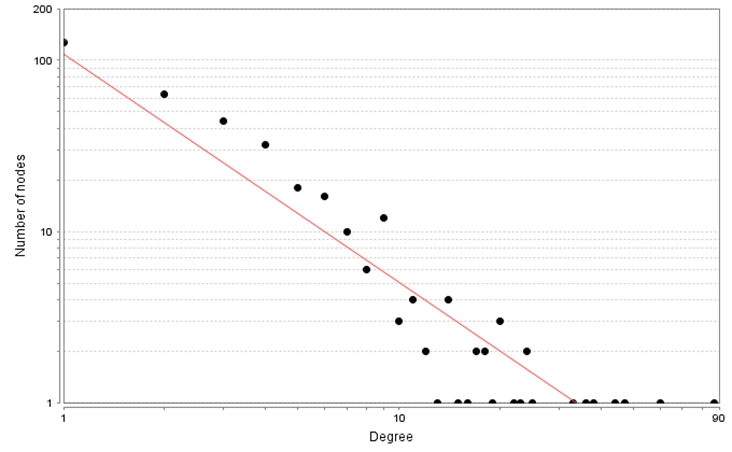

Supplement: S2 Fig — (TIF) [file pone.0191568.s002.tif]

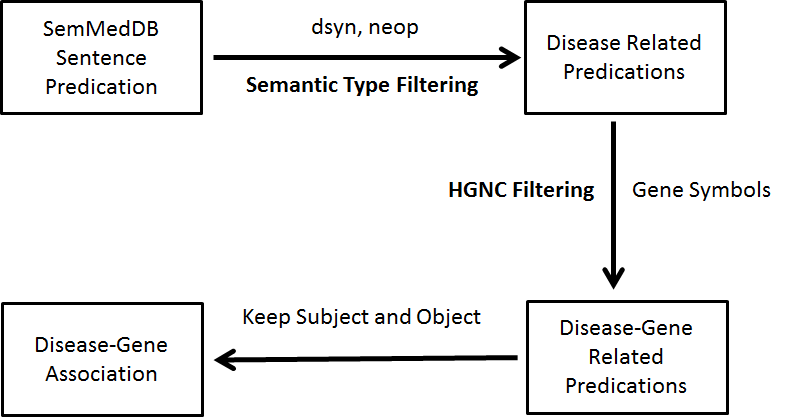

Supplement: S3 Fig — (TIF) [file pone.0191568.s003.tif]

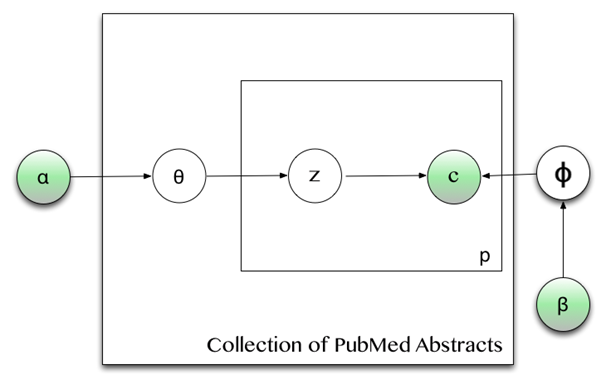

Supplement: S4 Fig — (TIF) [file pone.0191568.s004.tif]
