# Supplementary material for: Systematic identification of latent disease-gene associations from PubMed articles
Source: PLoS One. 2018 Jan 26;13(1):e0191568. doi: 10.1371/journal.pone.0191568 (PMC5786305; doi:10.1371/journal.pone.0191568)
Supplement: S6 File — (PDF) [file pone.0191568.s010.pdf]

INGENUITY
PATHWAY ANALYSIS

Analysis Name: case2 - 2017-12-11 01:10 PM
Analysis Creation Date: 2017-12-11
Build version: 460209M
Content version: 39480507 (Release Date: 2017-09-14)

Analysis Settings

Reference set: Ingenuity Knowledge Base (Genes Only)
Relationship to include: Direct and Indirect
Includes Endogenous Chemicals
Optional Analyses: My Pathways My List

Filter Summary:
Consider only relationships where
confidence = Experimentally Observed

(c) 2000-2017 QIAGEN. All rights reserved.

INGENUITY
PATHWAY ANALYSIS

Top Canonical Pathways

Name	p-value	Overlap
p53 Signaling	5.00E-26	27.0 % 30/111
Pancreatic Adenocarcinoma Signaling	1.12E-23	24.2 % 29/120
Aryl Hydrocarbon Receptor Signaling	1.50E-21	20.6 % 29/141
Prostate Cancer Signaling	2.51E-21	25.8 % 25/97
Bladder Cancer Signaling	3.66E-21	27.3 % 24/88

Top Upstream Regulators

Upstream Regulator	p-value of overlap	Predicted Activation
TP53	1.67E-63	
beta-estradiol	7.73E-59	
TGFB1	1.48E-57	
EGFR	3.84E-51	
phorbol myristate acetate	7.08E-46	

Top Diseases and Bio Functions

Diseases and Disorders

Name	p-value	#Molecules
Cancer	2.15E-24 - 2.87E-107	400
Organismal Injury and Abnormalities	2.17E-24 - 2.87E-107	402
Reproductive System Disease	1.77E-24 - 1.44E-80	323
Tumor Morphology	2.11E-25 - 9.00E-79	179
Respiratory Disease	2.54E-28 - 1.77E-69	199

Molecular and Cellular Functions

(c) 2000-2017 QIAGEN. All rights reserved.

INGENUITY PATHWAY ANALYSIS

Name	p-value	#Molecules
Cellular Development	1.88E-24 - 4.60E-116	314
Cellular Growth and Proliferation	1.88E-24 - 4.60E-116	308
Cellular Movement	4.59E-25 - 2.00E-114	269
Cell Death and Survival	2.17E-24 - 6.71E-105	319
Cell Cycle	4.28E-25 - 1.86E-62	191

Physiological System Development and Function

Name	p-value	#Molecules
Cardiovascular System Development and Function	1.27E-25 - 1.00E-72	185
Tissue Development	1.88E-24 - 2.76E-68	267
Organismal Development	3.30E-25 - 1.12E-67	280
Tissue Morphology	6.48E-26 - 1.92E-64	221
Embryonic Development	3.30E-25 - 5.76E-61	240

Top Tox Functions

Assays: Clinical Chemistry and Hematology

Name	p-value	#Molecules
Increased Levels of Alkaline Phosphatase	1.95E-02 - 5.50E-08	12
Increased Levels of Red Blood Cells	3.39E-05 - 3.39E-05	10
Increased Levels of LDH	3.81E-02 - 3.79E-05	6
Increased Levels of Albumin	1.46E-01 - 3.80E-04	4
Decreased Levels of Albumin	2.26E-01 - 4.65E-03	4

Cardiotoxicity

(c) 2000-2017 QIAGEN. All rights reserved.

INGENUITY PATHWAY ANALYSIS

Name	p-value	#Molecules
Cardiac Necrosis/Cell Death	7.59E-02 - 6.46E-24	44
Cardiac Enlargement	3.77E-01 - 7.44E-22	49
Cardiac Proliferation	2.11E-01 - 4.31E-15	21
Congenital Heart Anomaly	4.36E-01 - 4.10E-10	25
Cardiac Fibrosis	3.77E-01 - 2.69E-09	21

Hepatotoxicity

Name	p-value	#Molecules
Hepatocellular Carcinoma	1.12E-01 - 2.06E-47	108
Liver Hyperplasia/Hyperproliferation	1.29E-01 - 2.06E-47	242
Liver Proliferation	5.74E-02 - 7.66E-28	45
Liver Necrosis/Cell Death	3.39E-01 - 6.17E-21	36
Liver Damage	1.29E-01 - 1.34E-12	36

Nephrotoxicity

Name	p-value	#Molecules
Renal Necrosis/Cell Death	9.39E-02 - 7.66E-30	71
Renal Proliferation	7.59E-02 - 3.78E-17	40
Renal Inflammation	2.00E-01 - 1.26E-10	32
Renal Nephritis	2.00E-01 - 1.26E-10	32
Glomerular Injury	9.39E-02 - 1.47E-10	35

Top Networks

ID	Associated Network Functions	Score
1	Cancer, Organismal Injury and Abnormalities, Respiratory Disease	45
2	Cell Death and Survival, Cellular Development, Cellular Growth and Proliferation	38

(c) 2000-2017 QIAGEN. All rights reserved.

INGENUITY PATHWAY ANALYSIS

3	Cellular Movement, Cancer, Organismal Injury and Abnormalities	34
4	Cancer, Endocrine System Disorders, Organismal Injury and Abnormalities	29
5	Cell Signaling, Molecular Transport, Vitamin and Mineral Metabolism	29

Top Tox Lists

Name	p-value	Overlap	
Renal Necrosis/Cell Death	9.23E-38	13.0 %	71/547
Liver Proliferation	2.71E-31	19.0 %	45/237
p53 Signaling	3.28E-27	27.7 %	31/112
Increases Liver Hyperplasia/Hyperproliferation	8.07E-27	27.0 %	31/115
Cardiac Necrosis/Cell Death	1.71E-26	15.3 %	44/288

(c) 2000-2017 QIAGEN. All rights reserved.

INGENUITY PATHWAY ANALYSIS
