# Supplementary material for: Systematic identification of latent disease-gene associations from PubMed articles
Source: PLoS One. 2018 Jan 26;13(1):e0191568. doi: 10.1371/journal.pone.0191568 (PMC5786305; doi:10.1371/journal.pone.0191568)
Supplement: S7 File — (PDF) [file pone.0191568.s011.pdf]

INGENUITY®
PATHWAY ANALYSIS

Analysis Name: case3 - 2017-12-11 01:12 PM
Analysis Creation Date: 2017-12-11
Build version: 460209M
Content version: 39480507 (Release Date: 2017-09-14)

Analysis Settings

Reference set: Ingenuity Knowledge Base (Genes Only)

Relationship to include: Direct and Indirect

Includes Endogenous Chemicals

Optional Analyses: My Pathways My List

Filter Summary:

Consider only relationships where

confidence = Experimentally Observed

(c) 2000-2017 QIAGEN. All rights reserved.

Top Canonical Pathways

Name	p-value	Overlap
Th1 and Th2 Activation Pathway	6.30E-35	20.5 % 38/185
Th1 Pathway	1.98E-27	21.5 % 29/135
Th2 Pathway	2.01E-23	18.0 % 27/150
Crosstalk between Dendritic Cells and Natural Killer Cells	1.13E-19	22.5 % 20/89
Altered T Cell and B Cell Signaling in Rheumatoid Arthritis	1.44E-19	22.2 % 20/90

Top Upstream Regulators

Upstream Regulator	p-value of overlap	Predicted Activation
TNF	3.09E-63	
IL2	3.80E-54	
lipopolysaccharide	1.80E-52	
IL4	9.86E-52	
phorbol myristate acetate	1.08E-50	

Top Diseases and Bio Functions

Diseases and Disorders

Name	p-value	#Molecules
Inflammatory Response	6.66E-22 - 4.18E-79	187
Organismal Injury and Abnormalities	8.97E-22 - 4.18E-79	257
Cancer	8.97E-22 - 2.18E-60	246
Immunological Disease	1.28E-22 - 2.74E-57	202
Connective Tissue Disorders	4.77E-22 - 5.21E-57	127

Molecular and Cellular Functions

(c) 2000-2017 QIAGEN. All rights reserved.

INGENUITY PATHWAY ANALYSIS

Name	p-value	#Molecules
Cellular Development	8.86E-22 - 3.66E-85	206
Cellular Growth and Proliferation	8.28E-22 - 3.66E-85	216
Cell-To-Cell Signaling and Interaction	1.06E-22 - 3.73E-76	167
Cell Death and Survival	8.28E-22 - 6.24E-76	212
Cellular Function and Maintenance	1.34E-22 - 7.94E-70	169

Physiological System Development and Function

Name	p-value	#Molecules
Hematological System Development and Function	8.28E-22 - 1.47E-82	192
Tissue Morphology	8.57E-23 - 1.47E-82	182
Lymphoid Tissue Structure and Development	8.28E-22 - 8.68E-82	165
Embryonic Development	4.61E-22 - 3.52E-77	161
Hematopoiesis	8.28E-22 - 3.52E-77	133

Top Tox Functions

Assays: Clinical Chemistry and Hematology

Name	p-value	#Molecules
Increased Levels of AST	8.58E-02 - 1.04E-08	7
Increased Levels of Creatinine	4.70E-06 - 4.70E-06	7
Increased Levels of Red Blood Cells	6.83E-06 - 6.83E-06	9
Increased Levels of Hematocrit	3.15E-05 - 3.15E-05	8
Increased Levels of Blood Urea Nitrogen	6.84E-05 - 6.84E-05	4

Cardiotoxicity

(c) 2000-2017 QIAGEN. All rights reserved.

Name	p-value	#Molecules
Cardiac Necrosis/Cell Death	8.58E-02 - 4.22E-16	28
Cardiac Infarction	1.43E-01 - 1.45E-15	26
Cardiac Enlargement	1.00E00 - 1.43E-13	32
Cardiac Fibrosis	3.77E-02 - 6.88E-11	19
Cardiac Inflammation	2.16E-01 - 5.64E-09	15

Hepatotoxicity

Name	p-value	#Molecules
Liver Damage	7.40E-02 - 3.78E-31	48
Liver Inflammation/Hepatitis	4.01E-01 - 1.26E-30	48
Hepatocellular Carcinoma	7.40E-02 - 7.66E-26	64
Liver Hyperplasia/Hyperproliferation	2.93E-01 - 7.66E-26	140
Liver Necrosis/Cell Death	3.77E-02 - 1.14E-25	37

Nephrotoxicity

Name	p-value	#Molecules
Renal Inflammation	4.45E-01 - 3.74E-41	57
Renal Nephritis	4.45E-01 - 3.74E-41	57
Renal Necrosis/Cell Death	1.20E-01 - 8.17E-21	44
Renal Proliferation	8.58E-02 - 5.96E-12	28
Renal Damage	1.75E-01 - 1.50E-11	20

Top Networks

ID	Associated Network Functions	Score
1	Cell Cycle, Cellular Development, Cellular Growth and Proliferation	42
2	Cardiovascular Disease, Organismal Injury and Abnormalities, Connective Tissue Development and Function	30

(c) 2000-2017 QIAGEN. All rights reserved.

3	Hematological System Development and Function, Lymphoid Tissue Structure and Development, Tissue Morphology	28
4	Cellular Development, Cellular Growth and Proliferation, Hematological System Development and Function	27
5	Antimicrobial Response, Inflammatory Response, Cell-mediated Immune Response	21

Top Tox Lists

Name	p-value	Overlap	
Liver Necrosis/Cell Death	1.84E-25	12.2 %	37/304
Renal Necrosis/Cell Death	9.95E-23	8.0 %	44/547
Increases Renal Nephritis	3.20E-22	32.8 %	19/58
Increases Liver Damage	2.59E-18	16.3 %	22/135
Liver Proliferation	4.54E-18	11.4 %	27/237

(c) 2000-2017 QIAGEN. All rights reserved.
