# Supplementary material for: Systematic identification of latent disease-gene associations from PubMed articles
Source: PLoS One. 2018 Jan 26;13(1):e0191568. doi: 10.1371/journal.pone.0191568 (PMC5786305; doi:10.1371/journal.pone.0191568)
Supplement: S1 Table — (DOC) [file pone.0191568.s013.doc]

**S1 Table** . Top 10 topics based on their normalized posterior probability

| Topic | Normalized posterior probability |
| --- | --- |
| 115 | 0.02346 |
| 24 | 0.01541 |
| 94 | 0.01527 |
| 103 | 0.01395 |
| 136 | 0.01365 |
| 50 | 0.01332 |
| 112 | 0.01309 |
| 124 | 0.01223 |
| 43 | 0.01221 |
| 53 | 0.01212 |
