# Supplementary material for: Systematic identification of latent disease-gene associations from PubMed articles
Source: PLoS One. 2018 Jan 26;13(1):e0191568. doi: 10.1371/journal.pone.0191568 (PMC5786305; doi:10.1371/journal.pone.0191568)
Supplement: S3 Table — (DOC) [file pone.0191568.s015.doc]

**S3 Table**. Statistics of three disease ontologies

|  | Number of Classes | Number of Properties | Average Number of Children | Maximum Number of Children |
| --- | --- | --- | --- | --- |
| SNOMED-CT | 324,129 | 152 | 5 | 2,379 |
| DO | 11,280 | 15 | 4 | 101 |
| HPO | 15,381 | 0 | 3 | 31 |
