# Supplementary material for: Systematic identification of latent disease-gene associations from PubMed articles
Source: PLoS One. 2018 Jan 26;13(1):e0191568. doi: 10.1371/journal.pone.0191568 (PMC5786305; doi:10.1371/journal.pone.0191568)
Supplement: S4 Table — (DOC) [file pone.0191568.s016.doc]

**S4 Table .** Overview of the annotation results with three ontologies

| **Topics** | **Number of Diseases** | **SNOMED-CT Annotated Diseases** | **DO Annotated Diseases** | **HPO Annotated Diseases** |
| --- | --- | --- | --- | --- |
| Topic 115 | 472 | 392 (83%) | 112 (23.7%) | 41 (8.7%) |
| Topic 24 | 332 | 288 (86.7%) | 99 (29.8%) | 39 (11.7%) |
| Topic 94 | 403 | 353 (87.6%) | 104 (25.8%) | 44 (10.9%) |
| Topic 103 | 414 | 343 (82.9%) | 112 (27.1%) | 35 (8.5%) |
| Topic 136 | 386 | 343 (88.9%) | 105 (27.2%) | 49 (12.7%) |
| Topic 50 | 363 | 287 (79.1%) | 70 (19.3%) | 22 (6.1%) |
| Topic 112 | 332 | 286 (86.1%) | 66 (19.9%) | 24 (7.2%) |
| Topic 124 | 363 | 337 (92.8%) | 113 (31.1%) | 43 (11.8%) |
| Topic 43 | 380 | 328 (86.3%) | 86 (22.6%) | 32 (8.4%) |
| Topic 53 | 330 | 300 (90.9%) | 73 (22.1%) | 24 (7.3%) |
