# Supplementary material for: Systematic identification of latent disease-gene associations from PubMed articles
Source: PLoS One. 2018 Jan 26;13(1):e0191568. doi: 10.1371/journal.pone.0191568 (PMC5786305; doi:10.1371/journal.pone.0191568)
Supplement: S5 Table — (DOC) [file pone.0191568.s017.doc]

**S5 Table.** LDAKL score between top 10 topics (optimal values in bold)

|  | **115** | **24** | **94** | **103** | **136** | **50** | **112** | **124** | **43** | **53** |
| --- | --- | --- | --- | --- | --- | --- | --- | --- | --- | --- |
| **115** | **0.04** | 1.729 | 6.207 | 3.937 | 3.917 | 4.287 | 3.759 | 4.103 | 0.133 | 4.275 |
| **24** | 1.729 | **0.013** | 5.03 | 3.207 | 3.388 | 3.608 | 3.042 | 3.386 | 1.99 | 3.558 |
| **94** | 6.207 | 5.03 | **2.03** | 5.778 | 5.145 | 6.888 | 5.645 | 6.049 | 6.531 | 6.728 |
| **103** | 3.937 | 3.207 | 5.778 | **3.023** | 4.04 | 4.221 | 3.635 | 4.156 | 4.356 | 4.133 |
| **136** | 3.917 | 3.388 | 5.145 | 4.04 | **2.217** | 4.578 | 3.756 | 3.388 | 4.539 | 4.496 |
| **50** | 4.287 | 3.608 | 6.888 | 4.221 | 4.578 | **1.079** | 4.334 | 4.677 | 4.5 | 4.849 |
| **112** | 3.759 | 3.042 | 5.645 | 3.635 | 3.756 | 4.334 | **2.087** | 3.979 | 4.194 | 4.065 |
| **124** | 4.103 | 3.386 | 6.049 | 4.156 | 3.388 | 4.677 | 3.979 | **0.028** | 4.538 | 4.495 |
| **43** | 0.133 | 1.99 | 6.531 | 4.356 | 4.539 | 4.5 | 4.194 | 4.538 | **0.108** | 4.709 |
| **53** | 4.275 | 3.558 | 6.728 | 4.133 | 4.496 | 4.849 | 4.065 | 4.495 | 4.709 | **0.249** |
