# Supplementary material for: Systematic identification of latent disease-gene associations from PubMed articles
Source: PLoS One. 2018 Jan 26;13(1):e0191568. doi: 10.1371/journal.pone.0191568 (PMC5786305; doi:10.1371/journal.pone.0191568)
Supplement: S6 Table — (DOC) [file pone.0191568.s018.doc]

## S6 Table. List of diseases with highest node degree in the AD association network

| **Top Disease CUIs** | **Node degree in the network** |
| --- | --- |
| Parkinson disease | 87 |
| Alzheimer’s disease | 60 |
| neurodegenerative disorders | 47 |
| amyotrophic lateral sclerosis | 33 |
