# Supplementary material for: Systematic identification of latent disease-gene associations from PubMed articles
Source: PLoS One. 2018 Jan 26;13(1):e0191568. doi: 10.1371/journal.pone.0191568 (PMC5786305; doi:10.1371/journal.pone.0191568)
Supplement: S7 Table — (DOC) [file pone.0191568.s019.doc]

## S7 Table. Statistics of significant network motifs

| **Network Motif** | **Z-Score** | **P-Value** |
| --- | --- | --- |
|  | 13,425 | 0.001 |
|  | 758.82 | 0.001 |
|  | 24.017 | 0.003 |

Node shape: triangle – disease, square – gene.
